# Can ALS-Associated *C9orf72* Repeat Expansions Be Diagnosed on a Blood DNA Test Alone?

**DOI:** 10.1371/journal.pone.0070007

**Published:** 2013-07-19

**Authors:** Roger Pamphlett, Pak Leng Cheong, Ronald J. Trent, Bing Yu

**Affiliations:** 1 The Stacey Motor Neuron Disease Laboratory, Department of Pathology, Sydney Medical School, The University of Sydney, Sydney, Australia; 2 Department of Molecular and Clinical Genetics, Royal Prince Alfred Hospital and Sydney Medical School, The University of Sydney, Sydney, Australia; Louisiana State University Health Sciences Center, United States of America

## Abstract

Gene mutations that preferentially affect the CNS have been implicated in a number of neurological disorders. This leads to the possibility that a disease-causing mutation present only in CNS tissues could be missed if it were tested in a blood DNA sample only. The commonest mutation in amyotrophic lateral sclerosis (ALS) is an expansion of the hexanucleotide repeats of *C9orf72*. To find out if CNS-specific mutations of this gene could cause some cases of ALS we looked for differences in the size of *C9orf72* repeats between DNA from the CNS and from white blood cells (WBCs) of 38 sporadic ALS patients, using a repeat-primed PCR screening test. We also looked for differences in *C9orf72* repeats in WBC DNA from 6 ALS-discordant and 1 ALS-concordant monozygotic twins. Abnormally expanded *C9orf72* repeats were found in 13% of the ALS CNS samples, as well as in their paired WBC DNA. The 87% of ALS CNS samples with normal-sized *C9orf72* repeats had the same number of repeats in paired WBC samples. All ALS-discordant twins had the same normal numbers of WBC *C9orf72* repeats. Although previous work suggests some tissue mosaicism in *C9orf72* repeat size is probably present, this study indicates that this is not likely to be of sufficient magnitude to result in a normal *C9orf72* repeat length in blood but an abnormally expanded repeat length in the CNS. This suggests that a blood DNA test alone will usually be sufficient to make a diagnosis of *C9orf72* repeat-related ALS.

## Introduction

**Figure 1:**
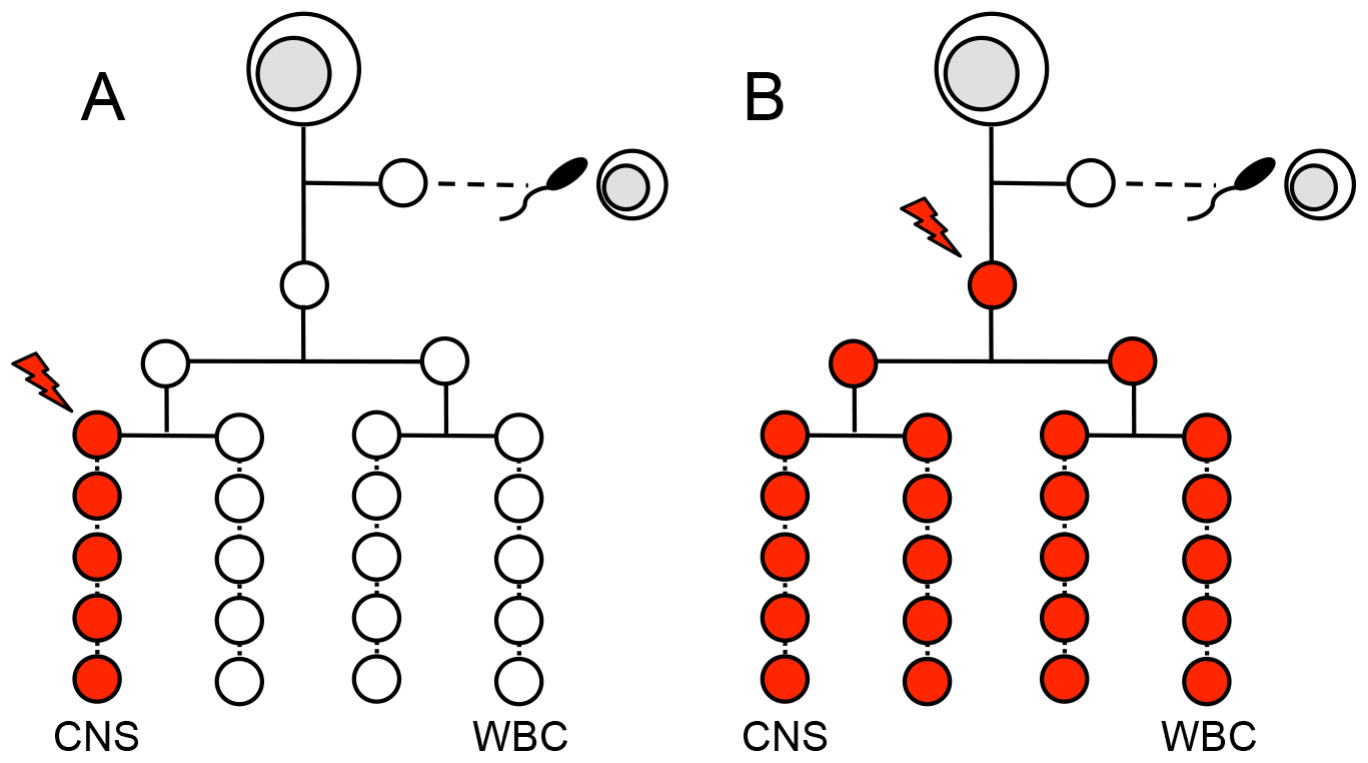
Early and late onset somatic mutations. In the human cell lineage, germline progenitor cells that produce the gametes (at the horizontal dashed line) are formed before the somatic cell lineages start dividing. The progenitor cells and their daughter cells for 4 somatic cell lines are shown. (A) Here a somatic mutation has occurred in a CNS progenitor cell, which is passed on to its daughter cells (red-filled circles) but which leaves the other cell lines intact. Comparing DNA from both the CNS and from white blood cells will reveal this somatic mutation. (B) The somatic mutation here has affected an early stem cell, so most or all of the somatic cell lines will contain the mutation. This mutation can be detected if one monozygous twin has a white blood cell mutation and the other does not.

In patients with sporadic amyotrophic lateral sclerosis (ALS) no commonly occurring cause can be found [1]. Genetic defects in white blood cell (WBC) DNA are present in only about 10% of these patients [2], and evidence for specific environmental agents causing the disease has been mixed [3]. One mechanism that needs to be considered in a sporadic disorder, when a blood test has failed to show a mutation, is a somatic mutation that involves the affected tissue selectively [4]. In early embryonic development, the germline progenitor cells separate from the dividing zygote before the somatic progenitors arise [5] (Fig. 1). This means that a somatic progenitor cell can sustain a mutation that does not affect the germline, and so is not passed on to the next generation. Somatic changes can be single nucleotide variants, structural variants, repeat expansions, or epigenetic changes. Somatic mutations underlie many cancers, and are found in a range of other diseases, including neurological disorders [6].

The number of different cell lineages affected by a somatic mutation depends on the timing of the mutation during embryonic development [4]. If a somatic mutation arises *late* in embryonic cell division, only one or a few cell lineages will carry the mutation (Fig. 1A). For example, if a mutation affects only a CNS progenitor cell, it will not be found in WBC DNA. To look for this type of somatic mutation, DNA from the tissue most affected by the disease needs to be compared with DNA from a tissue not affected by the disease. In the case of ALS, the most appropriate comparison would be between CNS and WBC DNA [7]. If the mutation arises *early* in embryonic cell division, it will involve most or all of the somatic cells (Fig. 1B), so an inter-tissue comparison is unlikely to identify the mutation. Here monozygous twins that are discordant for a disease can be informative, since a somatic mutation involving many cells lines in one of the twin members may be responsible for the disease arising in only that member [8], [9].

An abnormal expansion of *C9orf72* hexanucleotide repeat units is the commonest genetic variant associated with both familial and sporadic ALS [10], [11]. In an attempt to determine whether a blood DNA test is sufficient for the diagnosis of *C9orf72* repeat-related ALS, we compared normal and expanded repeat lengths between CNS and WBC samples from ALS patients, and in WBC samples between ALS-discordant monozygous twins. We found that abnormal increases in CNS *C9orf72* repeats lengths, with normal lengths in blood DNA, are unlikely to be present in ALS. Although the numbers of samples was relatively small, the results suggests that a routine blood test for *C9orf72* repeat expansions will usually be sufficient to make a diagnosis of *C9orf72* repeat-related ALS.

## Methods

### Ethics Statement

Informed written consent was obtained from each individual for their DNA to be used for research purposes. The project was approved by the Human Research Ethics Committee of the Sydney South West Area Health Service.

### Case Selection

Individuals selected for study were: (a) patients with ALS, either classical ALS or the lower motor neuron predominant variant of the disease, progressive muscular atrophy. Patients donated blood samples during life to the Australian Motor Neuron Disease DNA Bank and also pre-donated their brain and spinal cord for later *post mortem* removal under the “Using our Brains” donor program; (b) either ALS-discordant, ALS-concordant, or phenotypically normal monozygous twins, who donated blood samples to the Australian Motor Neuron Disease DNA Bank; zygosity was confirmed using AffyMetrix 6.0 arrays (AffyMetrix, Santa Clara, CA, USA) or microsatellite testing. No patient classified as sporadic ALS had any family history of ALS in reply to the questionnaire item “Has any member of your family (blood relatives) suffered from motor neuron disease”, or on a follow-up telephonic family history. Patients were not excluded from the study if there was a history of dementia in the family (a history of dementia in family members was noted, but no specific information about frontotemporal dementia was available). Further details of patients’ clinical histories, examinations and special investigations were made available by treating neurologists.

### Analysis of C9orf72 Hexanucleotide Repeats

Genomic DNA was extracted from: (a) WBCs using QIAmp blood kits (Qiagen, Hilden, Germany); (b) the cervical spinal cord and lateral frontal cerebral cortex from tissue that had been stored at −70°C, using a standard phenol chloroform method. Repeat-primed PCR was performed using primers as previously described [10]. PCR was undertaken with TopTaq Master Mix (Qiagen) to a final volume of 25 µL, with final concentrations of 1.2 µM 6-FAM labelled forward primer, 0.6 µM reverse primer, 1.2 µM anchor primer, 0.15 mM dNTP with GTP replaced by 7-deaza-dGTP (Roche, Basel, Switzerland), 7.2% DMSO, 1× Q-solution, 1.5 mM MgCl_2_ and 0.5 units of polymerase. 50 ng of DNA was used as template. The PCR annealing and extension ramp rates were adjusted to 0.5°C/s and 1.0°C/s respectively.

### Classification of Hexanucleotide Repeat Sizes

The *C9orf72* hexanucleotide repeat test is not accurate for sizes above 30 repeat units, i.e., a repeat of >100 will still appear as >30 repeats. For the purpose of this study, any hexanucleotide repeats above 30 in number were considered abnormal, and those 30 repeat units or less were considered normal [10]. Only one repeat number is provided because the method does not distinguish between the two alleles, so only the larger of the two (which superimposes over the lower repeat) is counted.

### Paired CNS-WBC Repeat Length Comparisons (Table 1)

For the CNS-WBC study, *C9orf72* repeats were measured in all available spinal cord samples; in 4 patients in whom spinal cord was not available, samples of frontal cerebral cortex were used. Since it was considered that only larger repeat sizes within the normal range were likely to be unstable, repeats were measured routinely only in WBC samples of patients who had either greater than 30 repeats, or repeats in the upper normal range (8 to 30 repeats), in their CNS samples; 3 random samples from the lower normal range in the CNS (1 to 7 repeats) were also measured in WBC samples to ensure the lower range was stable as presumed. Ages of individuals given in the Tables are those at the time of blood sampling.

### Twin Repeat Length Comparisons (Table 2)

For the twin study, *C9orf72* repeats were measured in DNA from WBCs in both members of all twin pairs.

### Cases and Controls

CNS samples were available from 38 sporadic ALS patients (mean age 65 y, SD 10 y, range 46–82 y, 27 males and 11 females) (Table 1). 36 patients had classical ALS and two had progressive muscular atrophy. Two of the CNS donor patients were also twins. One 79 year-old control male who donated blood and CNS tissue had no neurological disease.

**Table 1:** Size of *C9orf72* hexanucleotide repeats in CNS and WBC paired samples.

ID no.	Diagnosis	Age	Gender	CNS sample	*C9orf72* CNS repeat no.	*C9orf72* blood repeat no.
*Abnormal C9orf72 repeat sizes (N* >*30) in ALS*						
S01	ALS	69	Male	Spinal cord	>30	>30
S02	ALS	57	Male	Spinal cord	>30	>30
S03	ALS	59	Female	Spinal cord	>30	>30
S04	ALS	65	Female	Spinal cord	>30	>30
S05	ALS	59	Male	Frontal cortex	>30	>30
*Normal C9orf72 repeat sizes (N* ≤*30) in ALS*						
S06	ALS	81	Male	Spinal cord	18	18
S07	ALS	67	Male	Spinal cord	14	14
S08	ALS	60	Male	Spinal cord	12	12
S09	PMA	82	Female	Frontal cortex	12	12
S10	ALS	56	Male	Spinal cord	8	8
S11	ALS	56	Male	Spinal cord	8	8
S12	ALS	71	Male	Frontal cortex	8	8
S13	ALS	60	Female	Spinal cord	6	NT
S14	ALS	48	Male	Spinal cord	6	NT
S15	ALS	74	Female	Spinal cord	6	NT
S16	ALS	62	Male	Spinal cord	6	NT
S17	ALS	79	Male	Spinal cord	6	NT
S18	ALS¶	62	Female	Frontal cortex	6	6
S19	ALS	51	Male	Spinal cord	5	NT
S20	ALS	63	Male	Spinal cord	4	4
S21	ALS	78	Female	Spinal cord	4	NT
S22	ALS	46	Male	Spinal cord	3	NT
S23	ALS	53	Male	Spinal cord	3	NT
S24	ALS	66	Male	Spinal cord	3	NT
S25	ALS	77	Male	Spinal cord	3	NT
S26	ALS	60	Male	Spinal cord	3	NT
S27	ALS	78	Male	Spinal cord	3	NT
S28	ALS	54	Female	Spinal cord	1	NT
S29	ALS	78	Female	Spinal cord	1	NT
S30	ALS	57	Female	Spinal cord	1	1
S31	ALS	82	Male	Spinal cord	1	NT
S32	ALS	69	Male	Spinal cord	1	NT
S33	ALS	81	Male	Spinal cord	1	NT
S34	ALS	74	Female	Spinal cord	1	NT
S35	ALS	55	Male	Spinal cord	1	NT
S36	ALS	71	Male	Spinal cord	1	NT
S37	ALS	70	Male	Spinal cord	1	NT
S38	PMA§	52	Male	Spinal cord	1	NT
*Control individual*						
S39	Control	79	Male	Spinal cord	10	10

WBC repeats were not tested when CNS repeats were less than 8 in number (except for 3 samples).

¶ and §: twins as well as CNS donor (see also Table 2), NT: not tested.

Monozygous twin donors comprised: (a) 6 pairs of ALS-discordant twins (including a monozygous pair from triplets); (b) 1 pair of ALS-concordant twins; and (c) 6 pairs of control twins with no neurological disorders.

## Results

### Paired CNS-WBC Repeat Length Comparisons

1. Abnormally-enlarged *C9orf72* repeat expansions were found in 5 (13%) of the ALS CNS samples (S01-S05 in Table 1). In each of these patients the number of repeats in their paired WBC sample was also abnormally expanded.
2. Normal repeat unit lengths were found in 33 (87%) of the ALS CNS samples (S06-S38 in Table 1). In each of these patients where the WBC repeat number was measured it was the same as in the CNS DNA. There was no sign that repeat numbers in the higher normal range (*N* = 8–30) were unstable in CNS DNA.
3. The individual without any neurological disorder (S39 in Table 1) had the same normal number (*N* = 10) of *C9orf72* repeats in his CNS and WBC samples.

### Twin Repeat Length Comparisons

1. In all 6 ALS-discordant monozygous twin pairs (T01-T05 in Table 2), both twin members had *C9orf72* repeats of the same (normal) sizes. In the two monozygous members of the triplet (T06, one of whom was the ALS patient) each member had equal numbers (*N* = 6) of normal *C9orf72* repeats, but their dizygous sibling had a lower repeat number (*N* = 1).
2. Both of the members of one ALS-concordant monozygous twin pair (T07 in Table 2) had abnormally expanded *C9orf72* repeats. A blood sample from an older, ALS-unaffected sibling also showed an abnormally expanded *C9orf72* repeat.
3. The members in each of the 6 pairs of monozygous twins with no neurological disorders (T08-M13 in Table 2) had the same normal numbers of *C9orf72* repeats.

**Table 2:** Numbers of *C9orf72* repeats in white blood cell DNA of monozygous twins.

ID no.	Diagnosis	Gender	Age at blood sampling	Age at disease onset	Current status	*C9orf72* repeat no.
*ALS-discordant twins*						
T01-M1	ALS	Male	60	59	†ALS 61y	8
T01-M2	Control	Male	60	NA	Well 62y	8
T02-M1	ALS	Female	53	53	†ALS 55y	6
T02-M2	Control	Female	53	NA	Well 57y	6
T03-M1	ALS¶	Female	62	61	†ALS 63y	6
T03-M2	Control	Female	62	NA	Well 66y	6
T04-M1	ALS	Male	41	41	†ALS 42y	3
T04-M2	Control	Male	41	NA	Well 45y	3
T05-M1	PMA§	Male	52	52	†ALS 54y	1
T05-M2	Control	Male	52	NA	Well 58y	1
T06-M1♦	ALS	Female	69	68	ALS 71y	6
T06-M2♦	Control MZ	Female	69	NA	Well 71y	6
T06-M3♦	Control DZ	Female	69	NA	Well 71y	1
*ALS-concordant twins*						
T07-M1	ALS	Male	56	55	†ALS 57y	>30
T07-M2	ALS	Male	56	55	†ALS 62y	>30
*Control twins*						
T08-M1	Control	Female	38	NA	Well 44y	9
T08-M2	Control	Female	38	NA	Well 44y	9
T09-M1	Control	Female	56	NA	Well 63y	6
T09-M2	Control	Female	56	NA	Well 63y	6
T10-M1	Control	Female	68	NA	Well 70y	6
T10-M2	Control	Female	68	NA	Well 70y	6
T11-M1	Control	Male	54	NA	Well 59y	3
T11-M2	Control	Male	54	NA	Well 59y	3
T12-M1	Control	Male	74	NA	Well 80y	1
T12-M2	Control	Male	74	NA	Well 80y	1
T13-M1	Control	Female	80	NA	†leukemia 83y	1
T13-M2	Control	Female	80	NA	Well 85y	1

T: twin pair, M: member,

¶ and §: CNS donors as well (see also Table 1), NA: not applicable,

† : cause and age of death,

♦: triplet, MZ: monozygous, DZ: dizygous, PMA: progressive muscular atrophy.

### Patients Who were both CNS Donors and Twins

The two ALS patients who were tissue donors as well as monozygous twins (T03-M1, S18 and T05-M1, S38) had the same normal numbers of paired CNS and WBC *C9orf72* repeats, and the same WBC repeat number as their ALS-unaffected twin.

## Discussion

The major aim of this study was to see if *C9orf72* repeats are expanded preferentially in the CNS of ALS patients, and so would be missed if genetic analysis was undertaken on a blood sample only. The CNS tissues analysed were those most affected by ALS (the spinal cord and frontal motor cortex) to avoid sampling a CNS region that may not have contained the expansion due to site-specific mosaicism, since regional repeat mosaicism has been reported in other neurodegenerations such as spinocerebellar ataxia [12]. We did not find any differences in the sizes of *C9orf72* repeat lengths between matched CNS and WBC samples from sporadic ALS patients. It therefore appears unlikely that tissue mosaicism of *C9orf72* repeat lengths, with expanded repeats confined to the nervous system, can explain the mostly sporadic occurrence of ALS. These results indicate on the other hand that blood sampling is sufficient to make a diagnosis of *C9orf72*-related disease.

The repeat-primed method we used to screen for expanded *C9orf72* repeats is only accurate for up to about 60 repeats [10]. Numbers greater than 30 were considered pathological in this study, and fewer than 20 repeats normal. This range was based on a study of Finnish 402 ALS patients where 28.1% carried a repeat expansion, with an average size on repeat-primed PCR testing of 53 (range 30 to 71) repeats, compared to 478 controls where 99.6% had an average of 2 (range 0 to 22) repeats [10]. Although there is as yet no definitive cut-off point between normal and pathological repeat lengths, the cut-off used in the present study is that usually used for screening studies in *C9orf72*-associated disorders [13]. All of the repeats in our normal range were below 20 units in length, and none was present in the 20 to 30 border-zone range, so there appears to be no ambiguity between our normal and abnormal results.

This study does not rule out the possibility that mosaicism of *C9orf72* repeat lengths within the CNS may underlie the regional susceptibility of neurons to the *C9orf72* mutation. For example, the repeat lengths may be greater in motor regions of the brain and spinal cord than in sensory regions. To assess this, Southern blot methodology would be necessary to give an accurate measure of the repeat lengths [11]. Only recently has a reliable protocol for *C9orf72* repeat Southern blot hybridisation of different tissues, including peripheral blood, been reported [14]. Mentioned in this study were two patients who had *C9orf72* repeats measured in blood, cerebrum and cerebellum; abnormal repeat expansions were found in all tissues, but with different patterns of high molecular mass fragments [14]. This suggests that further exploration of the level of tissue mosaicism of *C9orf72* repeats could be of interest.

ALS is discordant in 90% of monozygous twins [15], which suggests that a non-germline variant such as a blastocyst cell somatic mutation may be responsible for the appearance of the disease in the affected twin. The main interest in our twin study was the possibility that a somatic difference in lengths of *C9orf72* repeat units could result in the appearance of ALS in one member of a monozygous twin pair. All of our ALS-discordant monozygous twins had normal numbers of *C9orf72* repeats, and the number of repeats was the same between the members of each twin pair. A *de novo* somatic difference in *C9orf72* repeat lengths cannot therefore explain why ALS affected only one member of each monozygous twin pair. This is in accord with a previous study showing how rarely copy number differences are present in WBC DNA between members of ALS-discordant monozygous twin pairs [16]. *C9orf72* repeats lengths were the same between all members of six control monozygous twin pairs, indicating that differences in these repeat lengths between any twins must be rare.

Both members of a monozygous twin pair who developed ALS within a few months of each other had abnormally-expanded *C9orf72* repeats. Their parents had died previously in their 60s, with no clinical features of ALS, though one of them had dementia. There was also a history of dementia in some other family members, and of paralysis in a great-grandparent. A phenotypically normal older sibling was found also to have expanded *C9orf72* repeats, so this appears to be familial *C9orf72* repeat-related disease.

In addition to the Australian twins that were studied in this project, we obtained Coriell Cell Repository WBC DNA from two pairs of sporadic ALS-discordant twins. Unfortunately, follow-up clinical details were not available for these twins and we cannot be sure if the initially unaffected twin developed ALS over time. These Coriell twins were therefore not included in this study. The findings are nevertheless of interest, because both 58 year-old male members of one twin pair had abnormally-expanded (>30) *C9orf72* repeats. This appears to be the only reported ALS-discordant twin pair where both twins have expanded *C9orf72* repeats. However, DNA is not available from other family members to ensure that this is not familial *C9orf72* repeat-related ALS. The other Coriell ALS-discordant twin pair of 35 year-old males both had normal and equal numbers of *C9orf72* repeats (*N* = 1).

Concerns about the stability of *C9orf72* repeats have been put forward previously [10]. These relate mostly to the suggestion that a degree of anticipation is present in some *C9orf72*-affected pedigrees, with the phenotype becoming apparent only in later generations [17]–[20]. We previously looked at the possibility that repeat instability might be responsible for *C9orf72*-related ALS in two patients in whom parental DNA was available, but in both of these trios one ALS-unaffected parent also had a pathologically-expanded repeat length [21]. In a further 41 trios in the latter study, each with an ALS-affected offspring, no difference in numbers of *C9orf72* repeats, all within the normal range, were seen. Furthermore, in the present study there was no sign that *C9orf72* repeat lengths in the high-normal range were unstable, since these did not differ between tissues or between monozygous twins. Overall, there appears to be little good evidence for *C9orf72* repeat instability, at least within the normal range of repeat lengths.

A limitation of the study is that the numbers of patients in both the blood-brain and twin cohorts were limited. This is because setting up a system of collecting ALS blood DNA samples during life and following up with later *post mortem* CNS donations is problematic. Similarly it is unusual to find live monozygotic ALS-affected monozygous twins who can both give blood DNA samples. The challenging nature of these collections is evidenced by the limited number of these samples that were collected over an 11 year period by Australia-wide blood DNA and CNS tissue donation Banks. The relatively small numbers means that these results with limited power must be treated with some caution, though the absence of even a single instance where differences between *C9orf72* repeat lengths were found between blood-CNS and between twins does suggest such differences are likely to be unusual.

Brain-situated somatic variants were first suggested as a possible cause of sporadic ALS in 1997, though in this study no mutations in the gene for Cu/Zn superoxide dismutase were found in ALS brain samples that were not present in blood [22]. Since then others have failed to find ALS brain-specific mutations involving either the length of androgen receptor triplet repeats [23], single nucleotide variants of coding and non-coding regions of *TARDP* [24], [25], or of all exons and the promoter of superoxide dismutase 1 [26]. ALS brain-situated genome-wide variants in chromosomal copy number [27] and in DNA methylation [28] have been reported, but these still require independent validation. Future exome and whole-genome sequencing of different tissues may be needed to reveal what role somatic mutations play in sporadic ALS.

The failure to find CNS-specific mutations in the commonest gene variation in ALS again raises the possibility that environmental agents may be playing a greater role in the disease than previously suspected. Twin studies have suggested the environmental contribution to ALS is in the order of 40% [15], and the age of onset in mutant *SOD1* families appears to be largely environmentally determined [29]. Furthermore, true sporadic ALS may be even more common than previously believed, since the occurrence of ALS in distant relatives may be by chance [30]. One meta-analysis arrived at a figure for FALS of 5% of total ALS patients [31], while another indicated that FALS due to penetrant mutations is likely to represent no more than 10% of total ALS patients [32]. Further investigations into environmental and gene-environmental factors in ALS are needed to clarify this issue.

In conclusion, our results indicate that *C9orf72* repeat expansions in the CNS are unlikely to arise either early or later in embryonic development in large enough numbers to cause diagnostic difficulties if blood DNA alone is being relied upon for testing. This means that clinicians can be confident that the great majority of *C9orf72* repeat mutations will be able to be diagnosed using blood-derived DNA.
